# Book Notices

**Published:** 1896-12

**Authors:** 


### Book Notices.

---

THE MEDICAL NEWS VISITING LIST FOR 1897.—Weekly (dated for 30 patients); Monthly (undated, for 120 patients per month); Perpetual (undated, for 30 patients weekly per year); and Perpetual (undated, for 60 patients weekly per year). The first three styles contain 32 pages of data and 160 of blanks. The 60-patient Perpetual consists of 256 pages of blanks. Each style in one wallet-shaped book, with pocket, pencil and rubber. Seal Grain Leather, $1.25. Philadelphia and New York: Lea Brothers & Co.

The Medical News Visiting List for 1897 has been thoroughly revised, and brought up to date in every respect. The text portion (32 pages) contains the most useful data for the physician and surgeon, including an alphabetical table of diseases, with the most approved remedies, and a table of doses. It also contains sections on examination of urine, artificial respiration, incompatibles, poisons and antidotes, diagnostic table of eruptive fevers, and the ligation of arteries. The classified blanks (160 pages) are arranged to hold records of all kinds of professional work, with memoranda and accounts.

When desired, a ready reference thumb-letter index is furnished, which is peculiar to this Visiting List, and which will save many-fold its small cost (25 cents) in the economy of time effected during a year.

---

THE PHYSICIANS' VISITING LIST (Lindsay & Blakiston's) for 1897.—Forty-sixth year of its publication. Price, for 25 patients per day or week, $1.00; for 50 patients per day or week, $1.25; for 75 patients per day or week, 2 vols., $2.00; for 100 patients per day or week, 2 vols., $2.25. P. Blakiston Son & Co., publishers, 1012 Walnut st., Philadelphia.

This visiting list is a great favorite with physicians, being compact and complete in arrangement, really a time saver. It has many valuable tables, including a calendar for 1897-1898, table of signs, the metric or French decimal system of weights and measures, table for converting apothecaries weights and measures into grams, dose table, and a new and complete table for calculating the period of utero-gestation. It has blank leaves for visiting list, memoranda, addresses of patients, addresses of nurses, accounts asked for, memoranda of wants, obstetric engagements, vaccination engagements, record of births, record of deaths, cash account, etc.

It is handsomely bound, has a pocket and pencil; in fact, is complete in every respect, and well deserves the great popularity it has attained.

